# A novel cytokine consisting of the p40 and EBI3 subunits suppresses experimental autoimmune arthritis via reciprocal regulation of Th17 and Treg cells

**DOI:** 10.1038/s41423-021-00798-2

**Published:** 2021-11-15

**Authors:** Seon-Yeong Lee, Su-Jin Moon, Young-Mee Moon, Hyeon-Beom Seo, Jun-Geol Ryu, A Ram Lee, Chae Rim Lee, Da-Som Kim, Yang-Mi Her, Jeong Won Choi, Seung-Ki Kwok, Sung-Hwan Park, Mi-La Cho

**Affiliations:** 1 The Rheumatism Research Center, The Catholic University of Korea, Seoul, South Korea; 2 Division of Rheumatology, Department of Internal Medicine, Seoul St. Mary’s Hospital, College of Medicine, The Catholic University of Korea, Seoul, South Korea; 3 Department of Biomedicine & Health Sciences, College of Medicine, The Catholic University of Korea, Seoul, South Korea; 4 Department of Medical Lifescience, College of Medicine, The Catholic University of Korea, Seoul, South Korea

**Keywords:** cytokine, p40-EBI3, Rheumatoid arthritis, Autoimmune disease, regulatory T cell, Autoimmunity, Cytokines

## Abstract

**Objective:**

The interleukin (IL)-12 cytokine family is closely related to the development of T helper cells, which are responsible for autoimmune disease enhancement or suppression. IL-12 family members are generally heterodimers and share three α-subunits (p35, p19, and p28) and two β-subunits (p40 and EBI3). However, a β-sheet p40 homodimer has been shown to exist and antagonize IL-12 and IL-23 signaling ^1^. Therefore, we assumed the existence of a p40-EBI3 heterodimer in nature and sought to investigate its role in immune regulation.

**Methods:**

The presence of the p40-EBI3 heterodimer was confirmed by ELISA, immunoprecipitation, and western blotting. A p40-EBI3 vector and p40-EBI3-Fc protein were synthesized to confirm the immunological role of this protein in mice with collagen-induced arthritis (CIA). The anti-inflammatory effects of p40-EBI3 were analyzed with regard to clinical, histological, and immune cell-regulating features in mice with CIA.

**Results:**

Clinical arthritis scores and the expression levels of proinflammatory cytokines (e.g., IL-17, IL-1β, IL-6, and TNF-α) were significantly attenuated in p40-EBI3-overexpressing and p40-EBI3-Fc-treated mice with CIA compared to vehicle-treated mice with CIA. Structural joint damage and vessel formation-related gene expression were also reduced by p40-EBI3 heterodimer treatment. In vitro, the p40-EBI3-Fc protein significantly suppressed the differentiation of Th17 cells and reciprocally induced CD4^+^CD25^+^Foxp3^+^ (regulatory T) cells. p40-EBI3 also inhibited osteoclast formation in a concentration-dependent manner.

**Conclusion:**

In this study, p40-EBI3 ameliorated proinflammatory conditions both in vivo and in vitro. We propose that p40-EBI3 is a novel anti-inflammatory cytokine involved in suppressing the immune response through the expansion of Treg cells and suppression of Th17 cells and osteoclastogenesis.

## Introduction

Rheumatoid arthritis (RA) is a type of immune-mediated chronic inflammatory arthritis characterized by synovial hypertrophy and progressive joint destruction. Several cytokines play pivotal roles in the pathogenesis of RA, thereby initiating, propagating, and maintaining inflammatory responses in the joints [1–4]. Members of the IL-12 family of cytokines have been shown to play important roles in RA. IL-12 family members (i.e., IL-12, IL-23, IL-27, IL-35, and IL-39) are heterodimeric type I cytokines produced by dendritic cells, macrophages, and B cells in response to antigenic stimulation [5–9]. IL-12 family members consist of one α-helix subunit (IL-23p19, IL-27p28, or IL-12p35) and one β-sheet subunit (IL-12p40 or EBI3) [10, 11].

IL-12, which is composed of the p35 and p40 subunits, was first identified in 1989 [12, 13]. IL-12 induces the differentiation of IFN-γ-producing Th1 cells from naïve CD4^+^ T cells through STAT4 phosphorylation via Jak2 and Tyk2 [14]. Both IL-12 and Th1 cells were initially implicated in the pathogenesis of RA [2]. IL-23, which consists of the common subunits p40 and p19, was discovered approximately 10 years after IL-12 was initially identified [15]. Notably, IL-23 is reportedly essential for the initiation and progression of inflammatory arthritis in a murine model of RA [16]. It is unclear whether this role involves the IL-12p40 subunit; however, unlike other IL-12 family cytokines, IL-12 and IL-23 exhibit mainly proinflammatory potential [2, 17].

Epstein-Barr virus-induced gene 3 (EBI3) is a subunit of IL-27, IL-35, and IL-39. In contrast to IL-12 and IL-23, IL-27, and IL-35 are related to the development of regulatory T (Treg) cells, which can suppress inflammation and control autoimmunity [18, 19]. IL-27 exhibits immunoregulatory potential in terms of limiting the intensity and duration of T cell responses and suppressing Th2 and Th17 responses through inhibition of the differentiation of the corresponding T cell subtypes from naïve T cells [20, 21]. IL-35, which consists of the EBI3 and IL-12p35 subunits, is produced by Treg cells; this novel inhibitory cytokine serves to maximize Treg cell activity [22]. IL-39, composed of the p19 α-subunit and EBI3 β-subunit, is the most recent addition to the IL-6/IL-12 family of cytokines. IL-39 remains a theoretical cytokine in humans [23].

The compositions and roles of five IL-12 family cytokines (IL-12, IL-23, IL-27, IL-35, and IL-39) in inflammation or autoimmunity have been characterized. We hypothesized the existence of an IL-12 family cytokine composed of the p40 and EBI3 subunits. In this study, we identified the existence of the p40-EBI3 heterodimer in nature. We then constructed an artificial p40-EBI3 protein and characterized its biological roles both in vitro and in a murine model of RA.

## Methods

### Animals and collagen-induced arthritis

Eight-week-old male DBA/1J (SLC, Shizuoka, Japan), C57BL/6 (The Jackson Laboratory, Bar Harbor, ME), BALB/c (Orient, Seoul, Korea), and p40-EBI3 transgenic (TG) mice were maintained in polycarbonate cages, with five animals per cage, in a specific pathogen-free environment. Mice were fed a standard mouse chow (Ralston Purina, St. Louis, MO, USA) and provided water ad libitum. All experimental procedures were reviewed and approved by the Animal Research Ethics Committee at the Catholic University of Korea. For the induction of arthritis, DBA1/J mice were immunized with type II collagen (CII) and complete Freund’s adjuvant (Chondrex, Redmond, WA, USA) via tail injection. At 2 weeks after the first immunization, the mice were boosted with CII and incomplete Freund’s adjuvant (Chondrex). At 1 week after collagen-induced arthritis (CIA) induction, a p40-EBI3 mock control vector was administered by electroporation; weekly hydrodynamic injection was then performed for 10 weeks, as described previously [24]. To analyze the effects of the p40-EBI3 protein, mice received intraperitoneal injection of the p40-EBI3 protein once per day after CIA induction. Beginning the day after primary immunization, three observers independently examined the severity of arthritis three times per week for up to 9–12 weeks. The severity of arthritis was recorded using the mean arthritis index with a scale of 0–4, as reported previously [25].

### P40-EBI3 transgenic mice

A *p40-*linker*-EBI3* fragment transgene, encoding p40-EBI3 fused to HA at the N-terminus, was inserted into the pcDNA3.1 HA plasmid. Microinjection of the transgene was performed to generate *p40*-*EBI3* transgenic mice on the C57BL/6 background (Macrogen Inc., Seoul, Korea). The presence of the transgene in founder mice was confirmed by polymerase chain reaction (PCR) using genomic DNA isolated from the tail as the template.

### Plasmid vector construction

To generate a plasmid for expression of the mouse p40-EBI3 protein, p40 and EBI3 constructs were designed with a 3XGGGGS linker connecting the proteins. The p40-linker-EBI3 fragment was synthesized by GenScript Corporation (Piscataway, NJ, USA) with codon optimization for expression in mammalian cells; it was then inserted into the HindIII and XbaI sites of the p3XFLAG-CMV vector. The vector encoded p40-EBI3 fused to FLAG [26].

### IL-12 receptor knockdown using siRNA

Transfection of IL-12 receptor-specific siRNA constructs was performed using the P3 Primary Cell 4D-nucleofector^TM^ X Kit (Lonza Bioscience, Basel, Switzerland), in accordance with the manufacturer’s recommendations, and the program DN-100 (Lonza Bioscience). Mouse splenocytes were stimulated with 0.5 μg/mL anti-CD3 for 12 h. The cells were then harvested and cotransfected with IL-12 receptor-specific (IL-12Rβ1 and WSX1 or IL-12Rβ1 and gp130; 100 nM) siRNAs (Santa Cruz Biotechnology, Santa Cruz, CA, USA) in accordance with the manufacturer’s protocol. Transfected cells were incubated in anti-CD3-coated plates and treated with the p40-EBI3 protein for 3 days.

### Construction of plasmid vectors for recombinant protein expression and protein purification

To generate a recombinant mouse p40-EBI3-Fc protein, the *p40*-linker-*EBI3* fragment was digested with *Hin*dIII and then inserted into the pAD11 plasmid. The resulting plasmid was transfected into Chinese hamster ovary (CHO)/dihydrofolate reductase (DHFR)^−/−^ cells (provided by Prof. Young Chul Sung of Pohang University of Science and Technology, Pohang, South Korea) using FuGENE®HD (Promega, Madison, WI, USA), which were selected in the absence of HT supplementation; methotrexate was used to enhance protein production. The cell culture supernatant was filtered through 0.45-μm membrane filters (Millipore, Billerica, MA, USA). The p40-EBI3-Fc protein was purified using a HiTrap Protein A HP 1-mL column (GE Healthcare, Little Chalfont, UK).

### T helper cell differentiation

Further details are provided in the Supplementary Methods.

### Flow cytometry

Stimulated T cells and IL-12 receptor-specific siRNA-transfected T cells were stained with anti-mouse CD4-peridinin-chlorophyll protein and anti-mouse CD25-allophycocyanin for 30 min at 4 °C. The cells were permeabilized and fixed with CytoPerm/CytoFix (BD Biosciences, San Diego, CA, USA) in accordance with the manufacturer’s protocol; further stained with anti-mouse Foxp3-phycoerythrin (PE) and/or anti-mouse IL-17-fluorescein isothiocyanate (FITC), anti-mouse IFN-γ-PE, and anti-mouse IL-4-PE (all from eBioscience, San Diego, CA, USA); and subjected to flow cytometric analysis (FACSCalibur; BD Biosciences). For p-STAT3 Y705 and S727 and p-STAT5 analysis, splenocytes were treated with p40-EBI3 (1 and 10 μg/mL) and IL-6 for 1 h; the cells were then stained with anti-mouse CD4-FITC. The cells were fixed and permeabilized with Lyse/Fix Buffer (BD Pharmingen, San Jose, CA, USA) and Perm Buffer II (BD Pharmingen). The permeabilized cells were stained with anti-p-STAT3 Y705-PE, anti-p-STAT3-S727-PE, or anti-p-STAT5-PE (BD Pharmingen) and subjected to flow cytometric analysis (CytoFLEX; Beckman Coulter, Fullerton, CA, USA).

### Mouse and human p40-EBI3 ELISAs

The concentrations of p40-EBI3 in culture supernatants and sera from patients were measured by ELISA with 96-well plates (Nunc, Roskilde, Denmark) coated with anti-mouse IL-27/IL-35 EBI3 (Cat. No. MAB18341; R&D Systems, Minneapolis, MN, USA) or anti-human IL-27/IL-35 EBI3 (Cat. No. BAF499; R&D Systems) and incubated overnight at 4 °C. After the overnight incubation, the plates were blocked with phosphate-buffered saline containing 1% bovine serum albumin and 0.05% Tween 20 for 2 h at room temperature. Purified p40-EBI3-Fc was used as the standard, and the protein was diluted twofold from 10 to 15.6 ng/mL. Cell culture supernatants were added to the plates and incubated at room temperature for 2 h. Subsequently, the plates were washed, biotinylated mouse IL-12/IL-23p40 (R&D Systems) or biotinylated human IL-12/IL-23p40 (Invitrogen) was then added, and the reaction mixtures were incubated for 2 h at room temperature. The plates were washed again and then incubated with ExtrAvidin-alkaline phosphate diluted 2000-fold (Sigma–Aldrich) for 2 h. Following an additional wash step, 50 μL of *p*-nitrophenyl phosphate disodium salt (Pierce Chemical Company) diluted in diethanolamine buffer at 1 mg/mL was added. The results were analyzed by determining the absorption at 405 nm (A_405_).

### CD4^+^ T cell proliferation assay

Splenocytes prepared from mice with CIA and p40-EBI3-Fc-treated mice were cultured with 100 μg/mL CII at 2 × 10^5^ cells/well in 96-well plates for 3 days. The cultured cells were treated with 0.5 μCi [^3^H] thymidine (GE Healthcare) during the final 16 h of the incubation. The incorporation of [^3^H] thymidine was measured using a Beta plate scintillation counter (PerkinElmer, Wellesley, MA, USA).

### IL-12Rβ1 and gp130 cotransfection into Ba/F3 cells

The mouse pro-B cell line BA/F3 was transfected with an IL-12Rβ1-GFP and gp130-GFP DNA vector (OriGene, Rockville, MD, USA) by using the P4 Primary Cell 4D-Nucleofector^TM^ X Kit (Lonza Bioscience), in accordance with the manufacturer’s recommendations, and the program DN-100 (Lonza Bioscience). The selection of transfected cells was achieved by sorting GFP-positive cells by FACS (BD FACSAria^TM^ Fusion cytometer; BD Biosciences)

### BAF3 cell proliferation

Transfected BA/F3 cells were cultured with p40-EBI3 (0.01–1 μg/mL) at 4 × 10^3^ cells/well in 96-well plates for 48 h. The cultured cells were treated with 0.5 μCi [^3^H] thymidine (GE Healthcare) during the final 16 h of the incubation. The incorporation of [^3^H] thymidine was measured using a Beta plate scintillation counter (PerkinElmer).

### Type II collagen-specific IgG and cytokine ELISAs

For CII-specific IgG, IgG1, and IgG2a analysis, serum was collected 70 days after the initial immunization. Flat-bottom 96-well plates were coated with a total of 40 μg/mL CII in phosphate-buffered saline (Nunc, Roskilde, Denmark) at 4 °C overnight. Serially diluted serum samples were applied and incubated at room temperature for 1 h. The plates were washed, and horseradish peroxidase (HRP)-conjugated goat anti-mouse IgG, IgG1, or IgG2a (Bethyl Laboratories, Montgomery, TX, USA) was added. HRP activity was measured using a tetramethylbenzidine solution (eBioscience). The results were analyzed by determining the absorption at 450 nm (A_450_). For analysis of TNF-α, IL-6, and IL-17 expression levels, plates were coated with anti-mouse TNF-α, IL-6, or IL-17 antibodies (all from R&D Systems) by incubation overnight at 4 °C. The plates were washed and then treated with a blocking solution for 2 h at room temperature. Cell culture supernatants and biotinylated mouse TNF-α, IL-6, and IL-17 were added; the reactions were then continued for 2 h. The plates were washed again, and ExtrAvidin-alkaline phosphate diluted 2000-fold(Sigma–Aldrich) was added. After 2 h of incubation, the samples were washed, 50 μL of *p*-nitrophenyl phosphate disodium salt diluted in diethanolamine buffer at 1 mg/mL was then added, and the results were analyzed by determining the A_405_.

### Immunoprecipitation and western blotting

Further details are provided in the Supplementary Methods.

### Real-time polymerase chain reaction

*Il-17*, *Foxp3*, *DC-STAMP, Atp6v0d2, Calcitonin receptor, Integrin β3, Mmp9, Trap, IL-12Rβ1, WSX1*, and *gp130* mRNA expression levels were determined by real-time PCR with SYBR Green I (Roche Diagnostic, Mannheim, Germany). Reaction mixtures were amplified on a LightCycler (Roche Diagnostic). Fluorescence curves were analyzed using LightCycler software v. 3.0. Expression levels were calculated and normalized relative to the values of a housekeeping gene (*GAPDH*) control.

### Immunohistochemistry

Fixed joint tissues embedded in paraffin were cut into 7-µm-thick sections, dewaxed using xylene, dehydrated through an alcohol series, and stained with hematoxylin and eosin, safranin O, or toluidine blue to detect proteoglycans. Endogenous peroxidase activity was quenched with methanol/3% H_2_O_2_. Immunohistochemistry was performed using a Vectastain ABC kit (Vector Laboratories, Burlingame, CA, USA). Sections were incubated with specific antibodies (i.e., anti-TNFα, anti-IL-1β, anti-IL-6, anti-IL-17, anti-RANKL, anti-VEGF, and anti-HIF-1α; all from R&D Systems) overnight at 4 °C. The tissue sections were then incubated with a biotinylated secondary antibody, followed by a streptavidin-peroxidase complex for 1 h. The final color product was developed using diaminobenzidine as the chromogen (DAKO, Carpinteria, CA, USA).

### Statistical analysis

Statistical analysis was performed using IBM SPSS Statistics, version 20 for Windows (IBM Corp., Armonk, NY, USA). Comparisons of multiple groups were performed by one-way ANOVA or the Kruskal–Wallis test; if a significant difference was observed among groups, Bonferroni’s or Dunn’s post hoc analysis was used to examine differences between specific groups. Comparisons of numerical data between two groups were performed with the nonparametric Mann–Whitney *U* test (two-tailed). In all analyses, *p* < 0.05 was considered to indicate statistical significance (*p < 0.05, ** *p* < 0.01, and *** *p* < 0.001). Data are presented as the means ± standard deviations (SDs).

## Results

### The p40-EBI3 protein is naturally present in immune cells

**Fig. 1:**
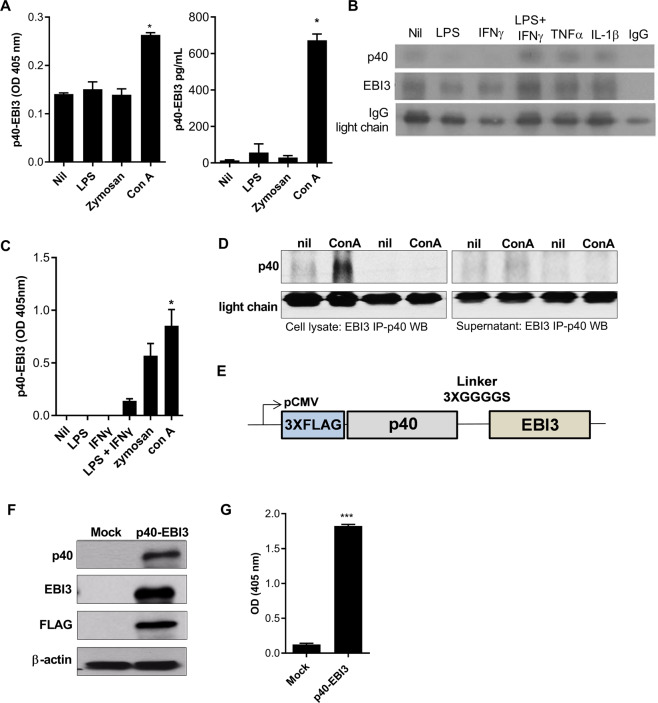
Identification of the p40-EBI3 protein in mice. **A** Splenocytes isolated from normal BALB/c mice were stimulated with LPS, zymosan, or Con A or were untreated (Nil). The expression levels of p40-EBI3 in culture supernatants were detected using ELISA and converted into OD (405 nm) and pg/mL values. **B** Detection of p40-EBI3 protein expression in splenic non-T cells by immunoprecipitation with western blot analysis. Splenic non-T cells isolated from WT BALB/c mice were stimulated under different conditions for 3 days. Prepared cell lysates were immunoprecipitated with an anti-EBI3 antibody and subjected to western blotting with an anti-p40 antibody. **C** CD11c^+^ splenic dendritic cells isolated from normal BALB/c mice were stimulated under different conditions. The expression levels of the p40-EBI3 protein in culture supernatants were determined by ELISA. **D** Detection of p40-EBI3 protein expression in splenic non-T cells by EBI3 immunoprecipitation with p40 western blot analysis. Splenic non-T cells isolated from WT BALB/c mice were stimulated with 5 μg/mL ConA for 3 days. The p40-EBI3 protein was detected in the cell lysates and culture supernatant. **E** Schematic map of the p40-EBI3 plasmid vector (details described in the Methods section). **F** HEK293 cells were transfected with the p40-EBI3 plasmid vector. The expression levels of p40, EBI3, and FLAG in transfected cells were determined by western blotting. **G** The expression levels of p40-EBI3 in the culture supernatants in **E** were determined by ELISA (*p < 0.05, **p < 0.01, ***p < 0.001).

We sought to confirm the presence of a heterodimeric cytokine composed of the p40 and EBI3 β-sheet subunits in immune cells. To identify the presence of the p40-EBI3 protein in mice, splenocytes isolated from WT BALB/c mice were cultured with or without LPS, zymosan, or concanavalin A (Con A). The concentrations of the p40-EBI3 protein in culture supernatants were measured by ELISA. The results showed that Con A enhanced the production of the p40-EBI3 protein in murine splenocytes (Fig. 1A). Immunoprecipitation with western blot analysis showed that the natural binding form of p40 and EBI3 existed naturally in splenic non-T cells isolated from WT BALB/c mice under stimulation with LPS, IFN-γ, or TNF-α (Fig. 1B). The natural p40-EBI3 protein was also detected in the culture supernatants of CD11c^+^ splenic dendritic cells (DCs), especially cell lysates and culture supernatants, under Con A stimulation, and the p40-EBI3 protein was detected by immunoprecipitation with western blot analysis (Fig. 1C, D). These findings indicated that a heterodimeric cytokine composed of the p40 and EBI3 subunits exists in DCs and non-T cells. To investigate the immunological role of this heterodimeric cytokine, the p40 and EBI3 subunit genes were inserted into a plasmid vector (Fig. 1E). This plasmid vector or a mock vector was transfected into HEK293 cells. In cells that had been transfected with the p40-EBI3 plasmid vector, high expression levels of p40 and EBI3 were confirmed by western blotting (Fig. 1F). The presence of p40-EBI3 in the culture supernatant of HEK293 cells transfected with the plasmid vector was also confirmed by ELISA (Fig. 1G).

### p40-EBI3 gene therapy ameliorates inflammatory arthritis in mice with CIA

**Fig. 2:**
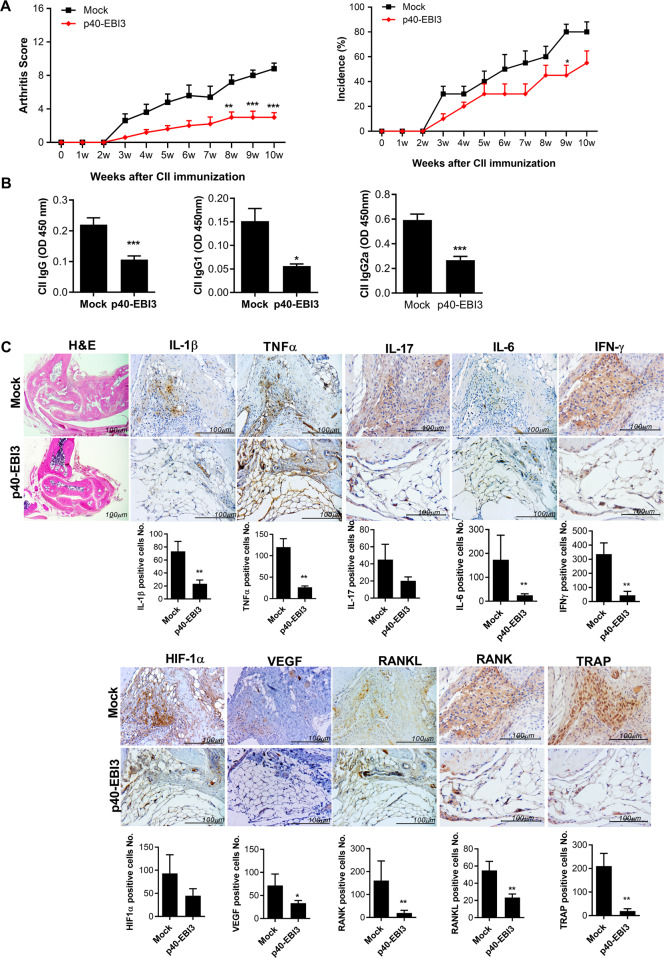
In vivo therapeutic effect of p40-EBI3 on the development of autoimmune arthritis. One week after immunization with type II collagen (CII), mice with CII-induced arthritis (CIA) were administered 100 μg of p40-EBI3 vector or FLAG vector (mock) in the muscles of both thighs by electroporation; they then received the p40-EBI3 vector or FLAG vector intravenously by hydrodynamic injection at weekly intervals until 10 weeks after CII immunization. **A** Representative results from one of three independent experiments are shown for the clinical scores (left) and incidences of arthritis (right) for each treatment group over time. **B** The concentrations of CII-specific IgG, IgG1, and IgG2a in sera from mice in each treatment group at 10 weeks after CII immunization were determined by ELISA. Bars show the mean ± SD of 10 mice per group. **C** At 10 weeks after the initial CII immunization, tissue sections were obtained from the ankle joints of mice with CIA and stained with hematoxylin and eosin (H&E) or antibodies specific for IL-1β, TNF-α, IL-17, IL-6, IFN-γ, HIF-1α, VEGF, RANKL, RANK, or TRAP. Representative images of antibody-positive cells (stained brown) are shown (top, scale bar = 100 μm); the results are depicted as the number of antibody-positive cells (mean ± SD) for five mice per group (bottom) (*p < 0.05, **p < 0.01, ***p < 0.001).

To investigate the immunological role of p40-EBI3 in arthritis-related inflammation, we performed gene therapy in mice with CIA, which constitute a murine model of RA. As in the mock vector-treated group, arthritis progressed steadily for 10 weeks after immunization with CII in p40-EBI3 vector-treated mice with CIA. However, both the clinical severity and incidence of arthritis of mice with CIA that had been treated with the p40-EBI3 vector were significantly reduced compared to those of mice in the mock vector-treated group (Fig. 2A). The levels of CII-specific IgG, IgG1, and IgG2a in the serum were also reduced by p40-EBI3 gene therapy (Fig. 2B). Several proinflammatory, proangiogenic, and osteoclastogenic mediators are reportedly involved in the development and progression of RA related to T cell immunity and osteoclastogenesis [27–31]. Therefore, we analyzed the expression levels of the above cytokines in the ankle joints of mice with CIA. Immunohistochemical analyses showed that the expression levels of inflammatory, angiogenic, and osteoclastogenic cytokines were significantly reduced by p40-EBI3 gene therapy (Fig. 2C). The population of tartrate-resistant acid phosphatase (TRAP)-positive multinucleated cells was reduced by p40-EBI3 gene therapy compared to mock vector treatment (Fig. 2C).

### Overexpression of p40-EBI3 suppresses arthritis severity and reduces the populations of germinal center B cells and inflammatory cytokine-expressing T cells

**Fig. 3:**
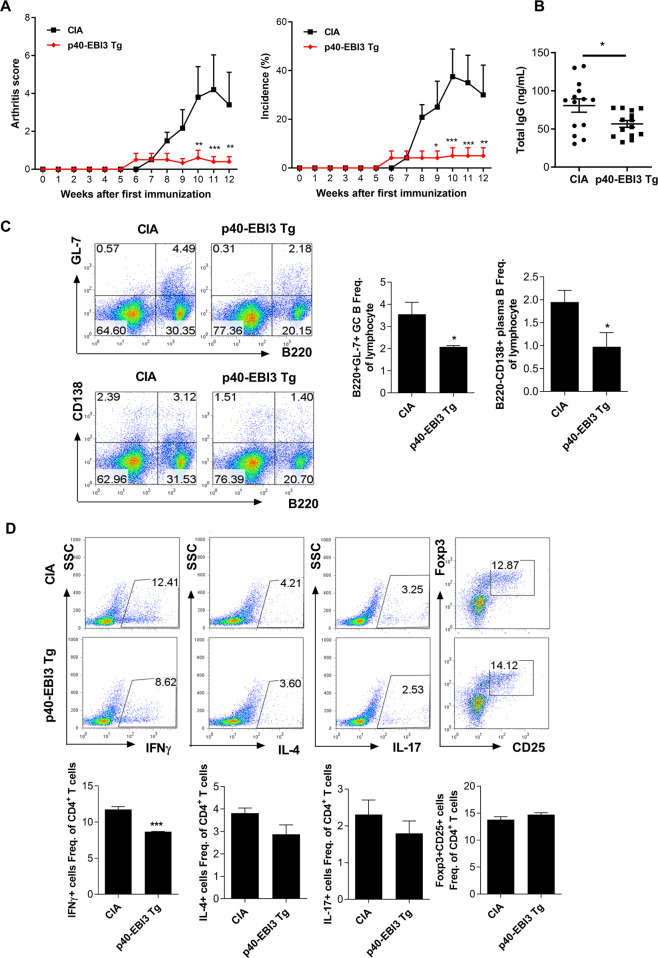
Attenuation of arthritis via regulation of the phenotype of immune cells in p40-EBI3 transgenic C57BL/6 mice achieved by microinjection of the transgene. Collagen-induced arthritis (CIA) was induced in WT or p40-EBI3 transgenic C57BL/6 mice as described in the Methods section. **A** Representative results from one of three independent experiments are shown for the clinical scores (left) and incidences of arthritis (right) for each treatment group over time. **B** The concentrations of total IgG in sera from mice in each group at 12 weeks after CII immunization were determined by ELISA. **C** Spleens were obtained from mice in each group at 12 weeks after CII immunization and analyzed by flow cytometry for B220^+^GL-7^+^ germinal center B cells and B220^−^CD138^+^ plasma cells. Representative flow cytometry plots from one of three independent experiments are shown (left); the results are depicted as the mean ± SD of three mice per group (right). **D** Mice were sacrificed at 12 weeks after CIA induction; the populations of IFN-γ^+^, IL-4^+^, IL-17^+^, and CD25^+^Foxp3^+^ cells among splenic CD4^+^ T cells were analyzed by flow cytometry. Representative flow cytometry plots from one of three independent experiments are shown (left); the results are depicted as the mean ± SD of three mice per group (right) (*p < 0.05, **p < 0.01, ***p < 0.001).

Next, we confirmed the immunoregulatory effect of the p40-EBI3 protein using p40-EBI3 transgenic (TG) mice. For this, CIA was induced in wild-type (WT) and p40-EBI3-TG C57BL/6 mice. Information about the generation and validation of p40-EBI3-TG mice is shown in Supplementary Figure 1. The results showed that the severity and incidence of arthritis were significantly lower in p40-EBI3 TG mice than in CIA mice (Fig. 3A). The serum level of total IgG was lower in p40-EBI3 TG mice than in CIA mice (Fig. 3B). To determine the cause of the reduction in the circulating immunoglobulin level, the populations of germinal center B cells and B220^−^CD138^+^ plasma cells were analyzed in the spleens of mice from each group using flow cytometry. Germinal center B cell formation has been shown to reflect pathological B cell function in mice with CIA [32]. Plasma cells have attracted attention as a treatment target in RA because they can produce disease-specific autoantibodies, such as anti-citrullinated protein antibodies, that are assumed to play a pivotal role in the development of RA [33]. We found that B220^+^GL-7^+^ germinal center B cells and B220^+^CD138^+^ plasma B cell populations were significantly smaller in p40-EBI3 TG mice than in CIA mice (Fig. 3C). Furthermore, the populations of inflammatory cytokine (IFN-γ, IL-4, and IL-17)-expressing splenic T cells were smaller in p40-EBI3 TG mice than in CIA mice, whereas the populations of these cells were only slightly increased among CD4^+^CD25^+^Foxp3^+^ Treg cells (Fig. 3D). Hence, we investigated the inhibitory effect of p40-EBI3 on osteoclast differentiation. Bone marrow-derived macrophages (BMMs) isolated from p40-EBI3 TG mice showed reduced osteoclast differentiation in vitro. TRAP and MMP9 mRNA expression levels were also significantly lower in cells from p40-EBI3 transgenic mice (Supplementary Figure 3). These results indicated that osteoclastogenesis was attenuated in the BMMs of p40-EBI3 TG mice compared to those of WT control mice.

### The p40-EBI3-Fc protein reduces proinflammatory cytokine levels and regulates the balance between Th17 and Treg cells

**Fig. 4:**
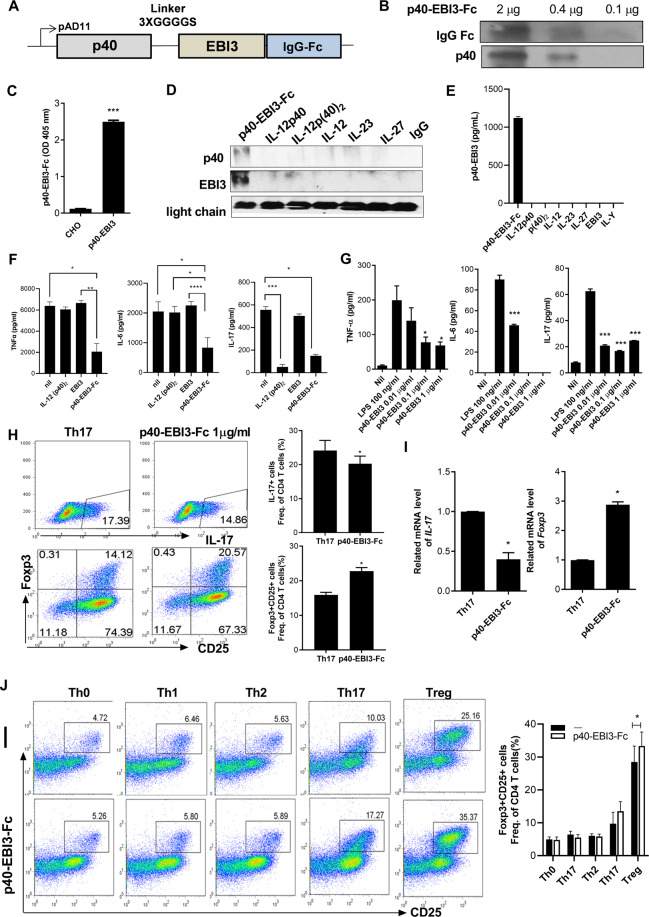
The p40-EBI3-Fc protein reduced proinflammatory cytokine levels and regulated the Th17/Treg cell balance. **A** Schematic map of the p40-EBI3-Fc expression vector used in CHO cells. **B** Western blotting for IgG Fc and IL-12p40 under treatment with different concentrations of p40-EBI3-Fc. **C** p40-EBI3-Fc protein expression was determined by ELISA. **D** Various proteins (p40-EBI3-Fc, recombinant IL-12p40, p(40)_2_, IL-12, IL-23, and IL-27) were immunoprecipitated with an anti-EBI3 antibody and subjected to western blotting with an anti-p40 antibody. **E** The expression levels of p40-EBI3-Fc and each recombinant protein were determined by p40-EBI3 ELISA. **F** The concentrations of TNF-α, IL-6, and IL-17 in the culture supernatants of cytokine-stimulated murine splenocytes were detected by ELISA. **G** The concentrations of TNF-α, IL-6, and IL-17 in the culture supernatants of LPS-stimulated murine splenocytes treated with or without the synthesized p40-EBI3-Fc protein at various concentrations (0.01-1 μg/mL). **H** Splenic CD4^+^ T cells from C57BL/6 mice were cultured under Th17 cell-inducing conditions in the presence or absence of the p40-EBI3-Fc protein (1 μg/mL). Three days later, the cells were stained with antibodies against CD4, IL-17, CD24, and Foxp3. Plot from a representative experiment showing the frequencies of IL-17^+^ and CD25^+^Foxp3^+^ cells among CD4^+^ T cells. Data are representative of four independent experiments with similar results. **I** IL-17 and Foxp3 mRNA expression levels were determined by real-time polymerase chain reaction. The values shown represent the mean ± SD. **J** Plot from a representative experiment showing the frequencies of CD25^+^Foxp3^+^ cells among CD4^+^ T cells under Th0, Th1, Th2, Th17, or Treg cell-inducing conditions. Data are representative of four independent experiments with similar results (*p < 0.05, **p < 0.01, ***p < 0.001).

We constructed an optimized p40-EBI3-Fc expression vector (Fig. 4A) and introduced it into the CHO cell line for protein expression and purification. Purified p40-EBI3-Fc protein was detected with antibodies specific for IgG-Fc or IL-12p40 (Fig. 4B). The results demonstrated the presence of p40 and EBI3 in purified protein from CHO cells. ELISA also confirmed the presence of our synthesized p40-EBI3 protein in the culture supernatants of CHO cells (Fig. 4C). Immunoprecipitation with western blot analyses showed that p40 and EBI3 existed in a purified p40-EB3-Fc complex compared to recombinant IL-12p40, IL-12p(40)_2_, IL-12, IL-23, and IL-27 (Fig. 4D), and the presence of p40-EBI3 was confirmed by ELISA (Fig. 4F). To verify the immunoregulatory properties of p40-EBI3-Fc, LPS-stimulated murine splenocytes were treated with purified p40-EBI3-Fc protein and compared to IL-12p(40)_2_- and EBI3-treated cells. The TNF-α, IL-6, and IL-17 levels in cells were reduced by treatment with the p40-EBI3-Fc protein in a dose-dependent manner (Fig. 4F, G). In addition, to investigate the mechanisms underlying the immunoregulatory properties of the p40-EBI3-Fc protein observed in vivo and in vitro, splenic CD4^+^ T cells isolated from C57BL/6 mice were cultured under Th17 cell-polarizing conditions in the presence or absence of the p40-EBI3-Fc protein (1 μg/mL); the levels of CD4^+^IL-17^+^ (mainly Th17) cells and CD4^+^CD25^+^Foxp3^+^ (mainly Treg) cells were determined by FACS analysis. The p40-EBI3-Fc protein reduced the Th17 cell population, whereas it enhanced the Treg cell population under the Th17 differentiation conditions (Fig. 4H). Real-time PCR results also showed that the level of IL-17 mRNA expression in cells was significantly reduced by the p40-EBI3-Fc protein, whereas the level of Foxp3 mRNA expression was enhanced (Fig. 4I). Subsequently, we examined whether the immunoregulatory function of p40-EBI3 was effective under other T cell subset-polarizing conditions. Surprisingly, the p40-EBI3 protein showed Treg cell induction only under Th17- or Treg-polarizing conditions but not under Th0-, Th1-, or Th2-inducing conditions, suggesting that the reciprocal regulation of Th17 and Treg cells is context dependent (Fig. 4J).

### The p40-EBI3-Fc protein suppresses Th17 cells via regulation of IL-12Rb1 and gp130 receptor signaling and transcription factor expression in vitro

**Fig. 5:**
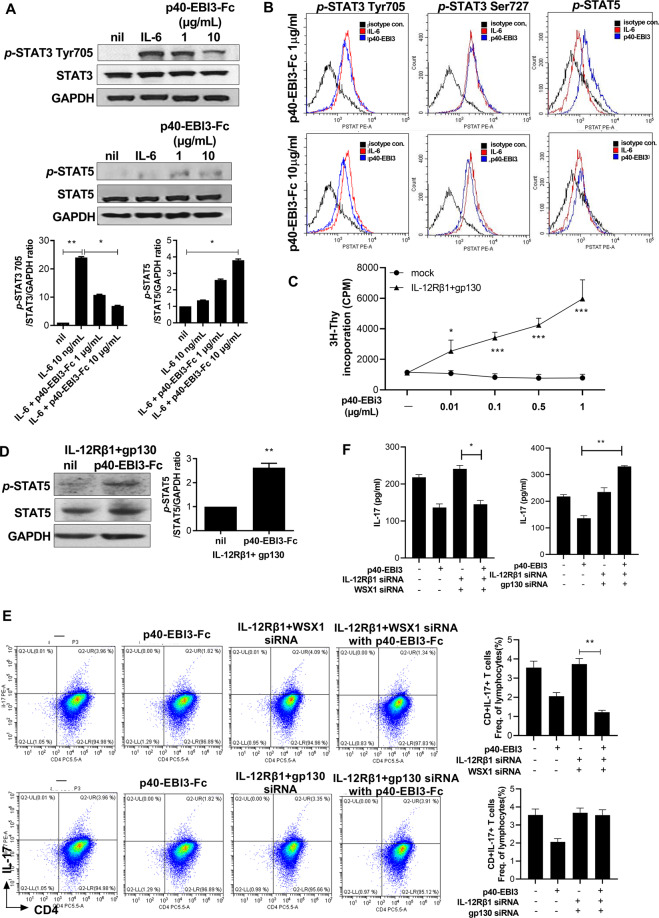
The p40-EBI3-Fc protein suppressed Th17 cells via regulation of IL-12Rb1 and gp130 receptor signaling. **A** Splenocytes from C57BL/6 mice were cultured with 10 ng/mL IL-6 in the presence or absence of the p40-EBI3-Fc protein (1 or 10 μg/mL). After 1 h, protein was isolated from the cultured cells. *p*-STAT3 Tyr705, STAT3, *p*-STAT5, STAT5, and GAPDH were analyzed by western blotting. Representative western blots from one of three independent experiments are shown (bottom panel); the results are depicted as the mean ± SD of three independent experiments per group (right). **B** Splenocytes isolated from C57BL/6 mice were stimulated with IL-6 in the presence or absence of the p40-EBI3-Fc protein (1 or 10 μg/mL) for 15 min. CD4^+^ T cells expressing pSTAT3 Tyr705, pSTAT3 Ser727, or pSTAT5 were then analyzed by flow cytometry. **C** A IL-12Rβ1 and gp130 overexpression vector was transfected into the Ba/F3 cell line using a P4 Primary cell 4D-nucleofector^TM^ X kit. The proliferation of Ba/F3 cells was determined with a [^3^H] thymidine incorporation assay under p40-EBI3-Fc (0.01–1 μg/mL) treatment. **D** IL-12Rβ1- and gp130-expressing Ba/F3 cells were stimulated with p40-EBI3-Fc for 1 h. *p*-STAT5, STAT5, and GAPDH in the cell lysates were analyzed using western blotting. Representative western blots from one of three independent experiments are shown; the results represent the mean ± SD of three independent experiments per group (right). **E**, **F** Alterations in IL-17 production in anti-CD3-stimulated murine splenic CD4^+^ T cells induced by p40-EBI3-Fc treatment and transfection with IL-12Rβ1-, gp130-, or WSX1-siRNA. Th17 cell expression was determined by FACS, and IL-17 expression was evaluated by ELISA. Bars show the mean ± SD of three independent experiments per group (*p < 0.05, **p < 0.01, ***p < 0.001).

STAT3 and STAT5 are pivotal transcription factors for Th17 and Treg cell differentiation, respectively. Accordingly, we investigated whether the p40-EBI3-Fc protein was associated with the regulation of phosphorylated STAT3 (*p*-STAT3) and phosphorylated STAT5 (*p*-STAT5). We cultured splenocytes from WT mice with IL-6 and the p40-EBI3-Fc protein. p40-EBI3-Fc enhanced the level of *p*-STAT5 but reduced the levels of phosphorylation at residues Tyr705 and Ser727 in STAT3 (Fig. 5A, B). These results suggested that the p40-EBI3-Fc protein reciprocally controlled the balance between Th17 and Treg populations via phosphorylation of STAT3 and STAT5. To determine the receptor responsible for mediating p40-EBI3 protein signaling, Ba/F3 cells [34] were transfected with IL-12Rβ1 and gp130 and then cultured in the presence of IL-3. GFP-positive Ba/F3 cells were selected by cell sorting and cultured with p40-EBI3 (0.01–1 μg/mL) at 4 × 10^3^ cells/well in 96-well plates for 48 h. Cell proliferation was analyzed to evaluate p40-EBI3-Fc-dependent growth. IL-12Rβ1- and gp130-expressing Ba/F3 cells proliferated in response to p40-EBI3 in a dose-dependent manner (Fig. 5C). Furthermore, the level of *p*-STAT5 was increased by p40-EBI3-Fc in IL-12Rβ1- and gp130-expressing Ba/F3 cells (Fig. 5D). To confirm the function of IL-12Rβ1 and gp130 as receptors for p40-EBI3, IL-12Rβ1-, gp130-, and WSX1-specific siRNAs were cotransfected into CD4 T cells from WT C57BL/6 mice. The results showed that IL-12Rβ1- and gp130-specific siRNA cotransfection attenuated the inhibitory effects of the p40-EBI3 protein on IL-17 production and Th17 cell expansion, whereas WSX1-specific siRNA cotransfection showed no such effect (Fig. 5E, F and Supplementary Fig. 2). These results suggested that IL-12Rβ1 and gp130 mediated signaling downstream of the p40-EBI3 protein.

### The p40-EBI3-Fc protein ameliorates arthritis and reduces osteoclast differentiation

**Fig. 6:**
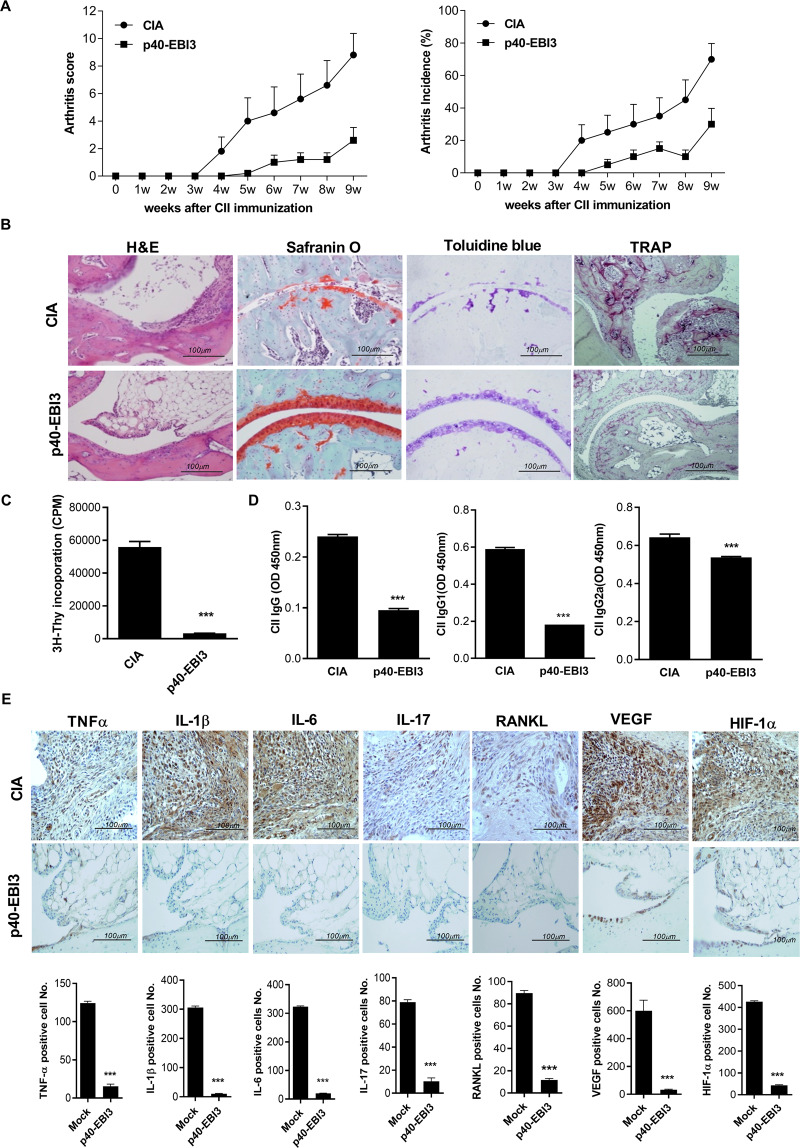
In vivo antiosteoclastogenic and anti-inflammatory properties of the p40-EBI3-Fc protein with respect to the development of collagen-induced arthritis (CIA). Mice with CIA received the p40-EBI3-Fc protein (2.5 mg/kg) or vehicle intraperitoneally daily after induction of CIA for 9 weeks. **A** Representative results from one of three independent experiments are shown for the clinical scores (left) and incidences of arthritis (right) for each treatment group over time. **B** At 63 days after initial CII immunization, tissues were obtained from the ankle joints of mice with CIA and stained with hematoxylin and eosin (H&E; original magnification, ×40), safranin O (original magnification, ×200), toluidine blue (original magnification, ×200), or tartrate-resistant acid phosphatase (TRAP; original magnification, ×200) to examine arthritis severity. **C** Isolated splenic CD4^+^ T cells from each group of mice were cultured for 3 days. T cell proliferation was determined by a [^3^H] thymidine incorporation assay. **D** The concentrations of CII-specific IgG, IgG1, and IgG2a in sera from mice in each group were determined by ELISA. Bars show the mean ± SD of four mice per group. **E** Tissue sections generated from the ankle joints of mice with CIA treated with or without p40-EBI3-Fc were stained with antibodies against TNFα, IL-1β, IL-6, RANKL, VEGF, and HIF-1α or with an isotype control. Cells stained with each antibody are shown in brown (upper). The numbers of positive cells were counted in high-power fields (magnification, ×400) with the aid of Adobe Photoshop software and averaged for three randomly selected fields per tissue section. Bars show the mean ± SD of three mice per group (*p < 0.05, **p < 0.01, ***p < 0.001).

We examined whether the p40-EBI3-Fc protein ameliorates the induction of arthritis. Animals that exhibited arthritis were administered intraperitoneal injections of the p40-EBI3-Fc protein for 9 weeks. The arthritis clinical score and incidence of arthritis were significantly reduced in mice treated with the p40-EBI3-Fc protein compared to those in the CIA group (Fig. 6A). Histological analysis of joints showed that the degrees of inflammatory cell infiltration and cartilage damage were milder in the p40-EBI3-Fc group (Fig. 6B). In addition, CD4^+^ T cell proliferation and CII-specific IgG, IgG1, and IgG2a levels were reduced in the p40-EBI3-Fc group (Fig. 6C, D). Immunohistochemical analysis showed that the levels of proinflammatory cytokines (e.g., TNF-α, IL-1β, IL-6, and IL-17) were reduced by administration of p40-EBI3-Fc. The RANKL, HIF-1α, and VEGF expression levels were also reduced (Fig. 6E). Subsequently, we investigated whether the populations of Th17 and Treg cells were altered by p40-EBI3-Fc treatment. Notably, the number of IL-17-expressing CD4^+^ T cells (mainly Th17 cells) was reduced by p40-EBI3-Fc treatment in mice with CIA, whereas the number of CD4^+^CD25^+^Foxp3^+^ Treg cells was marginally enhanced (Supplementary Fig. 4). The numbers of pSTAT3 Tyr705- and Ser727-expressing CD4^+^ T cells isolated from each group of mice were significantly reduced by p40-EBI3-Fc cytokine treatment, whereas the number of p-STAT5-expressing CD4^+^ T cells was enhanced (Supplementary Fig. 4). These results confirmed that p40-EBI3 could regulate proinflammatory cytokine expression and joint destruction.

### The p40-EBI3-Fc protein inhibits osteoclast differentiation

The inhibitory effect of the p40-EB3-Fc protein on osteoclastogenesis was confirmed in vitro. Mouse bone marrow cells were induced to undergo osteoclastogenesis by exposure to RANKL and M-CSF. Treatment with the p40-EB3-Fc protein suppressed osteoclastogenesis, but an Fc-control protein had no effect on osteoclast differentiation (Supplementary Fig. 5A). The TRAP-positive cell count indicated a dose-dependent reduction in osteoclast formation (Supplementary Fig. 5B). The expression levels of osteoclastogenesis-related genes, such as *DC-STAMP*, *ATP6VOD2*, *calcitonin receptor*, *integrin β3*, *MMP9*, and *TRAP*, were reduced by treatment with p40-EBI3-Fc. These findings indicated that p40-EBI3-Fc inhibited the osteoclastogenic pathway (Supplementary Fig. 5C).

### Elevated serum levels of p40-EBI3 in patients with RA and the clinical significance of p40-EBI3

To confirm the presence of p40-EBI3 in humans, we measured the serum levels of p40-EBI3 in healthy controls (HCs) and patients with autoimmune rheumatic diseases by ELISA. The median (interquartile range) serum optical density (OD) values of p40-EBI3 in HCs (*n* = 47; 0.14 [0.118–0.160]), patients with osteoarthritis (OA, *n* = 50; 0.129 [0.109–0.155]), and patients with systemic lupus erythematous (SLE, *n* = 60; 0.12 [0.100–0.170]) were similarly measured and showed no significant differences (Supplementary Fig. 6A). Interestingly, the OD values of p40-EBI3 in the serum of patients with ankylosing spondylitis (AS) or RA, which are representative types of inflammatory arthritis, were significantly higher (Supplementary Fig. 6A). The p40-EBI3 distribution by quartile revealed that CRP levels were significantly higher in patients with RA who exhibited a p40-EBI3 serum level in the upper quartile (>0.242 OD) compared to those with a level in the lower quartile (<0.128 OD) (Supplementary Fig. 6B). In addition, the serum level of p40-EBI3 showed a positive correlation with the RF titer (Supplementary Fig. 6C).

The in vitro and in vivo data consistently indicated the anti-inflammatory function of the p40-EBI3 cytokine. The higher serum concentration of p40-EBI3 in patients with high RA disease activity suggests that the p40-EBI3 cytokine is induced to relieve RA inflammation. In summary, the p40-EBI3 cytokine is presumed to relieve joint inflammation and inhibit osteoclastogenesis through Th17 cell inhibition and Treg induction via STAT3/STAT3 regulation (Supplementary Fig. 7).

## Discussion

In this study, we identified a novel IL-12 family cytokine that consists of the p40 and EBI3 subunits. Notably, only the proinflammatory IL-12 cytokines, IL-12 and IL-23, were previously shown to contain the p40 subunit. However, agents targeting p40 (e.g., ustekinumab) were shown to be effective against psoriasis and Crohn’s disease but exhibit inadequate efficacy in RA [35], which is a common autoimmune inflammatory disease. Thus, we suspected the existence of an unknown IL-12 cytokine containing the p40 subunit. IL-27 and IL-35, which include the EBI3 subunit, exhibit immunoregulatory properties mediated through Th17 cell inhibition and Treg induction, in contrast to IL-12 and IL-23. The present study was performed under the assumption that EBI3 may transmit anti-inflammatory signals and that an unidentified molecule may contain an EBI3 subunit and a p40 subunit.

Here, we confirmed the natural presence of the heterodimeric p40-EBI3 cytokine in both mice and humans. We confirmed the anti-inflammatory and immunoregulatory properties of this cytokine in vivo using gene therapy and transgenic mice. The antiarthritic effect of p40-EBI3 in vivo was associated with the inhibition of Th17 cells. Next, we generated a p40-EBI3-Fc fusion protein and confirmed its anti-inflammatory effects in vitro. To investigate whether this p40-EBI3-Fc fusion protein could be applicable as a novel therapeutic agent for RA, its anti-inflammatory efficacy was examined in mice with CIA, a murine model of RA. The results showed that the p40-EBI3-Fc fusion protein significantly attenuated both the incidence and severity of arthritis in mice with CIA. In addition, the extent of structural joint damage and the expression of inflammatory cytokines were markedly inhibited by p40-EBI3-Fc treatment. Transgenic mice carrying the optimized p40-EBI3 gene (i.e., p40-EBI3 Tg mice) also showed resistance to inflammatory bowel disease (data not shown).

Despite the in vitro and in vivo immunoregulatory activities of p40-EBI3, the serum CRP level was higher in patients with RA who exhibited a serum p40-EBI3 level in the upper quartile than in those with a level in the lower quartile. Furthermore, the serum level of p40-EBI3 in patients with RA showed a positive correlation with the autoantibody titer. Combined with the experimental results of the present study, these clinical data imply that the synthesis of p40-EBI3 may be induced to compensate for inflammation. The results of the present study may at least partially explain why agents targeting p40 (e.g., ustekinumab) have been ineffective in the treatment of RA.

Currently, the heterodimeric cytokine p40-EBI3 is the only member of the IL-12 cytokine family known to be composed of two different β-subunits. EBI3 has been identified as a hematopoietic receptor-like protein induced in B cells by Epstein-Barr virus infection. EBI3 is structurally related to p40 and presumed to associate with other cytokine subunits [36]. The α subunit of IL-12p35, IL-23p19, and IL-27p28 is similar to that of IL-6 and is characterized by a four-α-helix bundle [37]. These α subunits are linked to one of two β subunits (p40 for IL-12 and IL-23 and EBI3 for IL-27) that are structurally related to soluble IL-6 receptor alpha (sIL-6Rα) [38]. Thus, these heterodimers are analogous to complexes composed of a cytokine and soluble receptor, and the ability to secrete the cytokine-like subunit depends on its association with a receptor-like subunit [9, 15].

IL-35 signaling in Tregs is transduced via combinations of IL-12Rβ2/gp130, IL-12Rβ2/IL-12Rβ2, and gp130/gp130 receptor, none of which have been clearly identified as the high-affinity receptor [39]. Additionally, gp130 is known for its capacity to transduce signals, especially signaling initiated by IL-6, a major cytokine involved in inflammation. The complex of IL-6 and IL-6R binds to the ubiquitously expressed receptor subunit gp130, which forms a homodimer and thereby initiates intracellular signaling via the JAK/STAT and MAPK pathways [40, 41]. p40-EBI3 complex signaling was transduced via a combination of the receptors IL-12Rb1 and gp130. Generally, p40 binds exclusively to IL-12Rb1 [42, 43], while EBI3 binds to WSX1 or gp130 [44]. In recent studies, gp130 signaling activity has been reported [45, 46]. It was confirmed that IL-12Rb1 and gp130 signaling could increase STAT5 activity while also inhibiting STAT3. The gp130 signaling induced by cytokines containing the EBI3 subunit was investigated in terms of the induction of inflammation and anti-inflammatory signals, depending on the cells and disease studied. The p40-EBI3 complex increased STAT5 expression, inhibited Th17 cells, and induced Tregs through IL-12Rb1 and gp130 receptor signaling.

Additionally, disulfide-linked p40-p40 homodimers are present in patients with multiple sclerosis [47]. Notably, the p40-p40 homodimer shows immunoregulatory activity via competition with heterodimers, antagonizing IL-12 signaling and Th1 cell expansion [48]. We previously reported the suppressive effect of the IL-12p40 homodimer [(p40)_2_] in a murine model of RA; recombinant (p40)_2_ prevented the development of arthritis and reduced the production of inflammatory cytokines [49].

In this study, we demonstrated that p40-EBI3 suppressed inflammatory arthritis *via* regulation of Treg cell expansion. We previously reported that the regulation of Th17 cells and Tregs is important in the treatment of RA because an imbalance between Th17 and Treg cells contributes to the development and progression of RA. Activation of STAT5 requires the maintenance of high Foxp3 expression and Treg expansion [50]. We observed that p40-EBI3 upregulated the generation of CD4^+^CD25^+^Foxp3^+^ Tregs through STAT5 activation. The p40 monomer enriches CD4^+^Foxp3^+^ T cells in bovine myelin basic protein (MBP)-primed splenocytes. In addition, p40 induces differentiation into Tregs under Th1 or Th17 differentiation conditions [51]. Furthermore, we previously reported the suppressive effect of (p40)_2_ in a murine model of RA; recombinant (p40)_2_ prevented the development of arthritis through reciprocal regulation of Th17 cells and Tregs. STAT3 is crucial for Th17 cell differentiation, whereas STAT5 functions as an inhibitor of Th17 cell differentiation. We observed that p40-EBI3 activated STAT5. Accordingly, p40-EBI3 also regulated the upregulation of Foxp3+ Tregs.

This is the first study to demonstrate the presence of a novel heterodimeric cytokine composed of two different β-subunits, p40 and EBI3, with immunoregulatory properties. To the best of our knowledge, this is the first study to identify the natural presence of the p40-EBI3 heterodimer in the blood of mice and humans. The anti-inflammatory effect of p40-EBI3-Fc treatment in a murine model of autoimmune arthritis suggests its potential use in novel treatments for inflammatory autoimmune diseases, including RA.

## Supplementary information

Supplementary Methods and figure
