# Supplementary material for: A novel cytokine consisting of the p40 and EBI3 subunits suppresses experimental autoimmune arthritis via reciprocal regulation of Th17 and Treg cells
Source: Cell Mol Immunol. 2021 Nov 15;19(1):79–91. doi: 10.1038/s41423-021-00798-2 (PMC8752814; doi:10.1038/s41423-021-00798-2)
Supplement: Supplementary file 1 — Supplementary Methods and figure [file 41423_2021_798_MOESM1_ESM.docx]

# Supplementary Methods

## T helper cell differentiation

Isolated CD4^+^ T cells (5 × 10^5^) from 6-week-old DBA1/J mice were incubated under different conditions to stimulate Th0, Th1, Th2, Th17, and Treg cells. Anti-CD3 (0.5 μg/mL; BD Biosciences) and anti-CD28 (1 μg/mL; BD Biosciences) antibodies were used to differentiate T helper cells. IL-12 (10 ng/mL; R&D Systems) and neutralizing antibodies to IL-4 (5 μg/mL) were added to stimulate the differentiation of Th1 cells. For Th2 cell differentiation, T cells were treated with IL-4 (10 ng/mL) and neutralizing antibody to IFN-γ (5 μg/mL). TGF-β (1 ng/mL; PeproTech, Rocky Hill, NJ, USA) and IL-6 (20 ng/mL), as well as neutralizing antibodies to IFN-γ (5 μg/mL) and IL-4 (5 μg/mL), were added for Th17 cell differentiation. TGF-β (1 ng/mL) and neutralizing antibodies to IFN-γ (5 μg/mL) and IL-4 (5 μg/mL) were added for differentiation of Treg cells. All recombinant and neutralizing antibodies were purchased from R&D Systems.

## Immunoprecipitation and western blotting

For immunoprecipitation, 5 μg of anti-EBI3 antibody (Santa Cruz Biotechnology, Dallas, TX, USA) or normal rat antiserum (control; Santa Cruz Biotechnology) was preincubated for 10 minutes with 10 μL of Dynabeads protein A solution (Dynal, Hamburg, Germany) at room temperature. Dynabeads protein A–antibody complexes were washed three times using binding buffers consisting of 0.1 M Na phosphate and 0.01% Tween 20. Aliquots of 100 μg of protein extracted from LPS (100 ng/mL; Sigma-Aldrich), IFNγ (25 ng/mL; R&D Systems), TNFα (20 ng/mL; R&D Systems), IL-1β (20 ng/mL; R&D Systems), ConA (5 μg/mL; Sigma-Aldrich) or ConA culture supernatant-stimulated non-T cells from WT BALB/C mice were incubated (with rotation) with Dynabeads protein A- anti-EBI3 antibody complex at room temperature for 10 minutes. For IP-WB of IL-12 family cytokines, 500 ng/mL of p40-EBI3-Fc, IL-12p40 (R&D Systems), p(40)_2_ (R&D Systems)_,_ IL-12 (R&D system), IL-23 (R&D Systems) or IL-27 protein (R&D Systems) was incubated with Dynabeads protein A-anti-EBI3 antibody complex. The complex was washed with phosphate-buffered saline and the Dynabeads-antibody-antigen complex was suspended in NuPAGE LDS sample buffer for 10 minutes at 70°C. The EBI3 antibody complex protein was separated by 10% sodium dodecyl sulfate (SDS) polyacrylamide gel electrophoresis and transferred onto nitrocellulose membranes (Amersham Pharmacia Biotech, Uppsala, Sweden). The membranes were incubated with primary antibodies to p40 (Santa Cruz Biotechnology) overnight at 4°C. Signals of primary antibody binding were detected using anti-rabbit HRP-conjugated secondary antibody (Santa Cruz Biotechnology). For p40-EBI3 western blotting analysis, purified p40-EBI3-Fc from CHO cell culture supernatant was subjected to 10% SDS polyacrylamide gel electrophoresis and transferred onto nitrocellulose membranes. The membranes were incubated with specific antibodies to p40 (R&D Systems), EBI3 (Santa Cruz Biotechnology), FLAG (OriGene), and β-actin (Santa Cruz Biotechnology). For *p*-STAT3 and *p*-STAT5 western blotting, whole splenocytes from C57BL/6 WT mice were cultured with IL-6 (10 ng/mL) and p40-EBI3-Fc (1 or 10 μg/mL) for 1 h. The lysate protein was separated by 10% SDS polyacrylamide gel. The membranes were incubated with specific antibodies to *p*-STAT3 Tyr705, STAT3, *p*-STAT5, STAT5 (all from Cell Signaling Technology, Danvers, MA, USA) or GAPDH (Abcam, Cambridge, UK). For IL-12 receptor western blotting, lysate protein of IL-12Rβ1, WSX1 or gp130 siRNA-transfected T cells were incubated with specific antibodies to IL-12Rβ1 (Santa Cruz Biotechnology), WSX1 (Abcam), gp130 (Santa Cruz Biotechnology) or GAPDH (Abcam). Signals of primary antibody binding were detected using anti-goat or anti-rabbit HRP-conjugated secondary antibodies as appropriate.

## *Ex vivo* and *in vitro* osteoclastogenesis

For *ex vivo* osteoclastogenesis experiments, bone marrow-derived macrophages (BMMs) obtained from mice were isolated from murine tibias and femurs by flushing the bone marrow cavity with α-modified Eagle’s medium (MEM; Invitrogen, Carlsbad, CA, USA). Cells were centrifuged and exposed to ACK buffer at room temperature for 30 s to remove red blood cells, then incubated with α-MEM containing penicillin/streptomycin and 10% FBS for 12 hours to separate floating and adherent cells. Floating cells were collected, suspended in α-MEM and counted, then seeded into 48-well plates at 1 × 10^5^ cells per well and cultured with α-MEM in the presence of 10 ng/mL recombinant human M-CSF (R&D Systems) for 3 days to form macrophage-like osteoclast precursor cells. After 3 days, nonadherent cells were washed out and adherent cells were used as BMMs. These osteoclast precursor cells were then cultured in the presence of 10 ng/mL recombinant human M-CSF and 50 ng/mL soluble recombinant human RANKL (PeproTech) for 4 days to generate osteoclasts. Differentiated osteoclasts were visualized by TRAP staining.

## Confocal microscopy

For confocal staining, 7-µm-thick splenic tissue sections were stained with FITC-conjugated anti-CD4, PE-conjugated anti-IL-17, PE-conjugated anti-pSTAT3 Ser727, PE-conjugated anti-pSTAT3 Tyr705, PE-conjugated anti-pSTAT5, PE-conjugated anti-CD4, allophycocyanin-conjugated anti-CD25, and FITC-conjugated anti-Foxp3 (all from eBioscience, San Diego, CA, USA). Stained sections were analyzed using a Zeiss microscope (LSM 510 Meta; Carl Zeiss, Oberkochen, Germany) at ×400 magnification.

## Patient population and ethics statement

Serum samples (IRB: HC11TISI0067) were obtained from HCs (*n* = 47), and from patients with OA (*n* = 50), SLE (*n* = 60), AS (*n* = 49), and RA (*n* = 136) who visited the outpatient department of the Division of Rheumatology, Bucheon St. Mary’s Hospital, Catholic University of Korea, between January 2016 and September 2018. All patients with RA who met the American College of Rheumatology criteria for RA (1), and 50 patients with OA who met the American College of Rheumatology criteria for OA (2), were included in this study. Forty-three healthy volunteers were included as controls. Serum samples were stored at −80°C until analysis. The levels of p40-EBI3 were measured in individual serum samples. This study was approved by the institutional review board of Bucheon St. Mary’s Hospital and performed in accordance with the tenets of the Declaration of Helsinki. All participants provided written informed consent to take part in the study.

## Clinical data and measurement of inflammatory markers

All patients were followed up to obtain clinical data, including age, sex, disease duration, erythrocyte sedimentation rate, C-reactive protein (CRP) level, and rheumatoid factor (RF) titer. RF titer was measured by particle-enhanced immunoturbidimetric assay; a level > 15 U/mL was considered positive. The CRP level was measured using an immunonephelometry method; values > 5 mg/L were considered positive.

# Supplementary Figure and legends


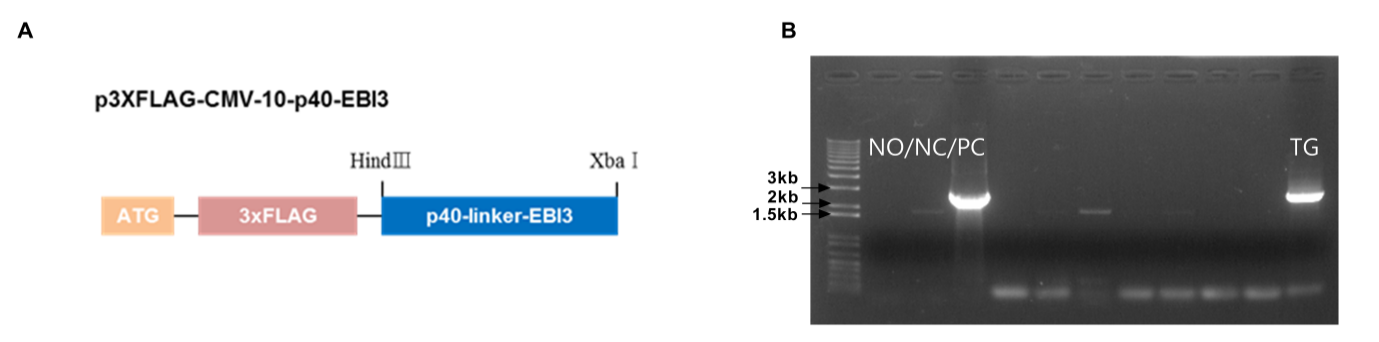


**Supplementary Figure 1.** Information for the vector of the p3XFLAG-CMV-10-TE TG mouse. (A) DNA purification for microinjection was completed by double-cutting the p3XFLAG-CMV-10-p40-EBI3 plasmid with NdeI / SfiI. (B) p40-EBI3 Tg mouse genotyping results. NO, no template; N/C, negative control; P/C, positive control.


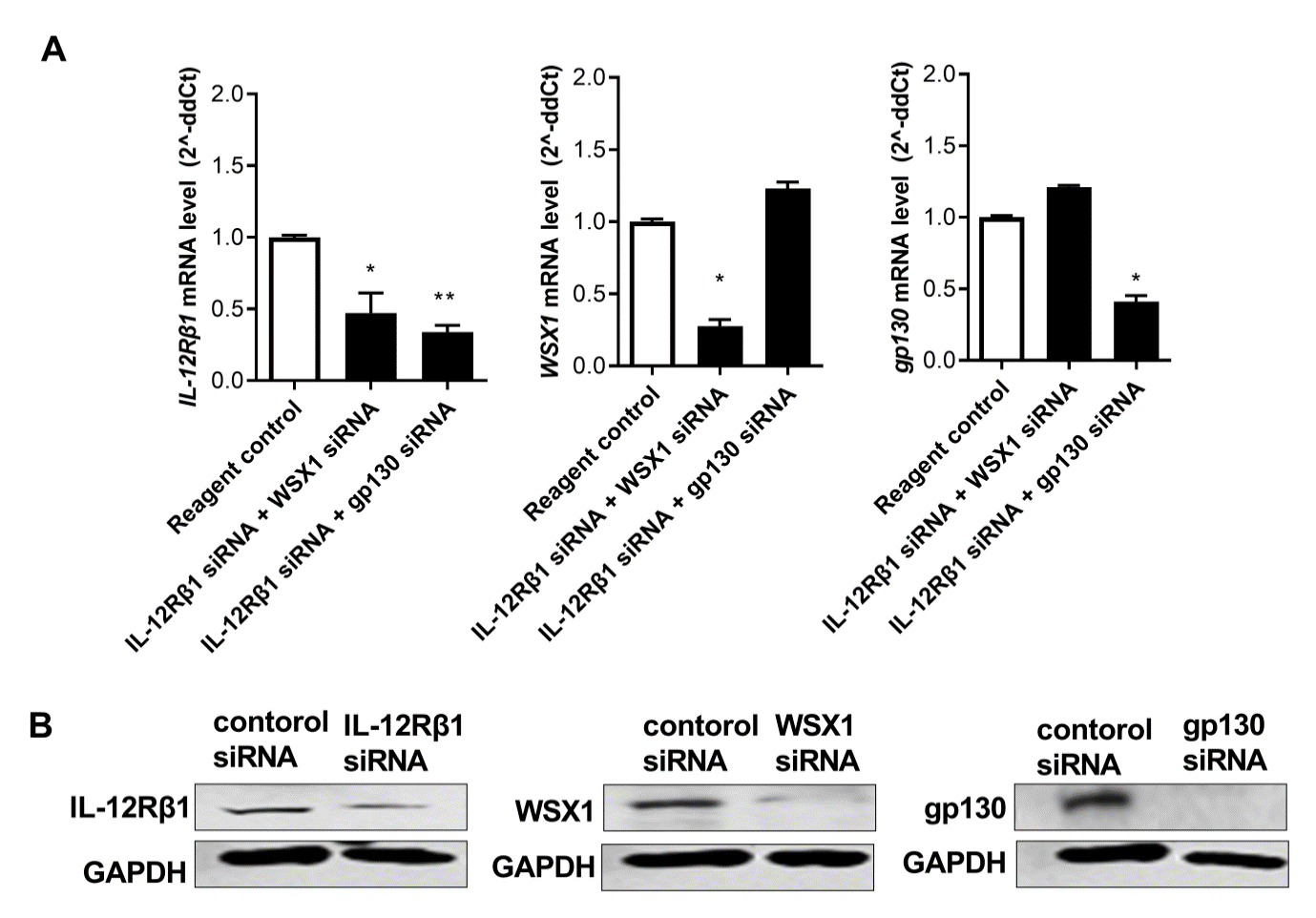


**Supplementary Figure 2.** Receptor siRNA reduced IL-12Rβ1, WSX1 and gp130 expression. (A) Alterations in IL-12Rβ1, WSX1 and gp130 gene expression in anti-CD3-stimulated murine splenic CD4^+^ T cells by transfection with IL-12Rβ1-, gp130-, or WSX1-siRNA. (B) siRNA-transfected T cells were determined in IL-12Rβ1, WSX1 and gp130 by Western blotting (**p* < 0.05, ***p* < 0.01).


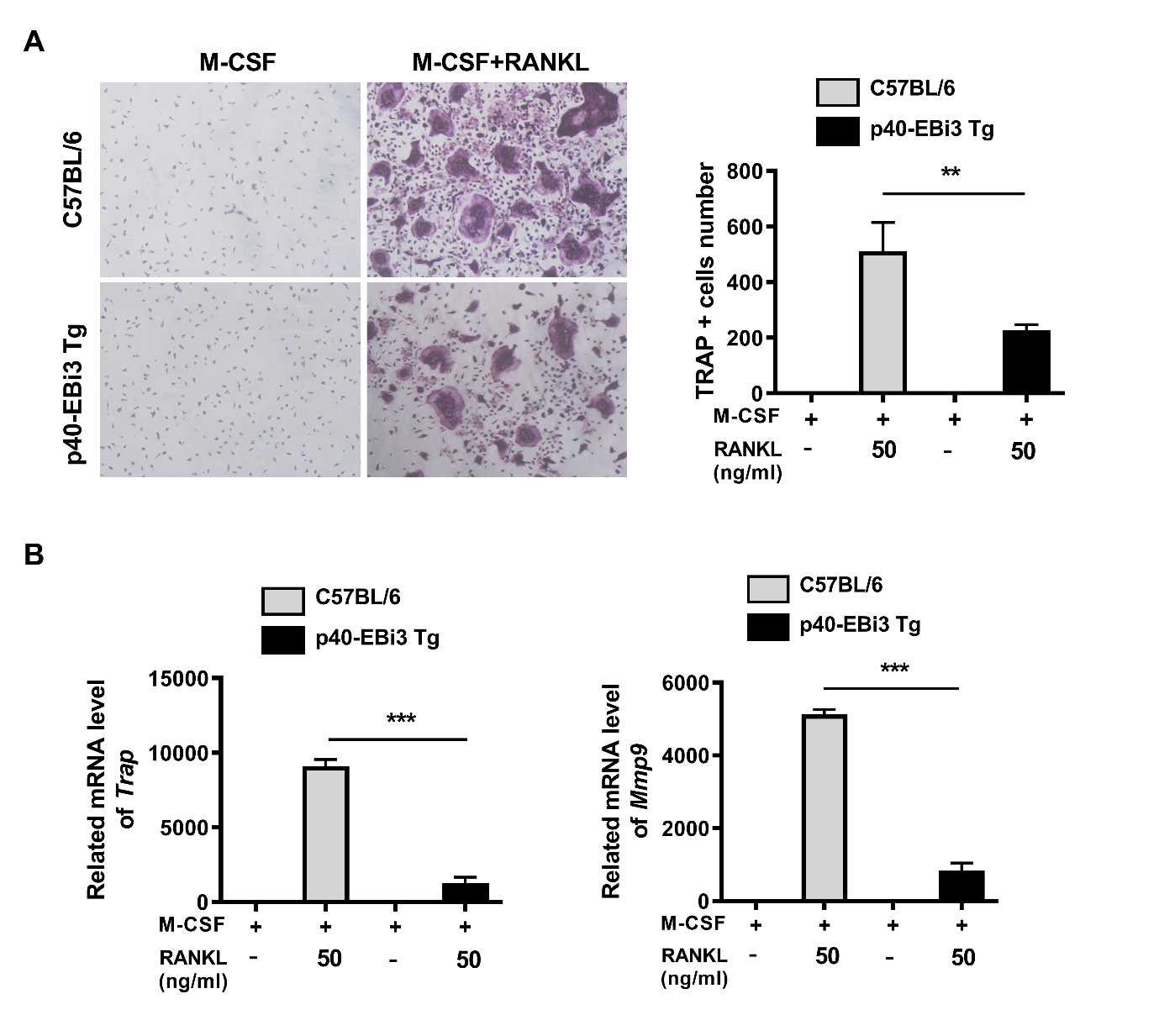


**Supplementary Figure 3.** BMMs isolated from p40-EBI3 transgenic mice showed significantly attenuated osteoclastogenesis *in vitro*. A. Osteoclast precursor cells from BMMs of WT C57BL/6 or p40-EBI3-transgenic C57BL/6 mice were cultured in the presence of macrophage colony-stimulating factor (M-CSF) (10 ng/mL), alone or together with RANKL (50 ng/mL). After 4 days, cells were stained with TRAP (representative images are shown). Numbers of multinucleated TRAP^+^ cells were determined. Twelve independent experiments were performed. B. mRNA expression levels of TRAP and MMP9 were determined by real-time PCR. Data are presented as the mean ± SD of three independent experiments (***p* < 0.01, ****p* < 0.001).

**
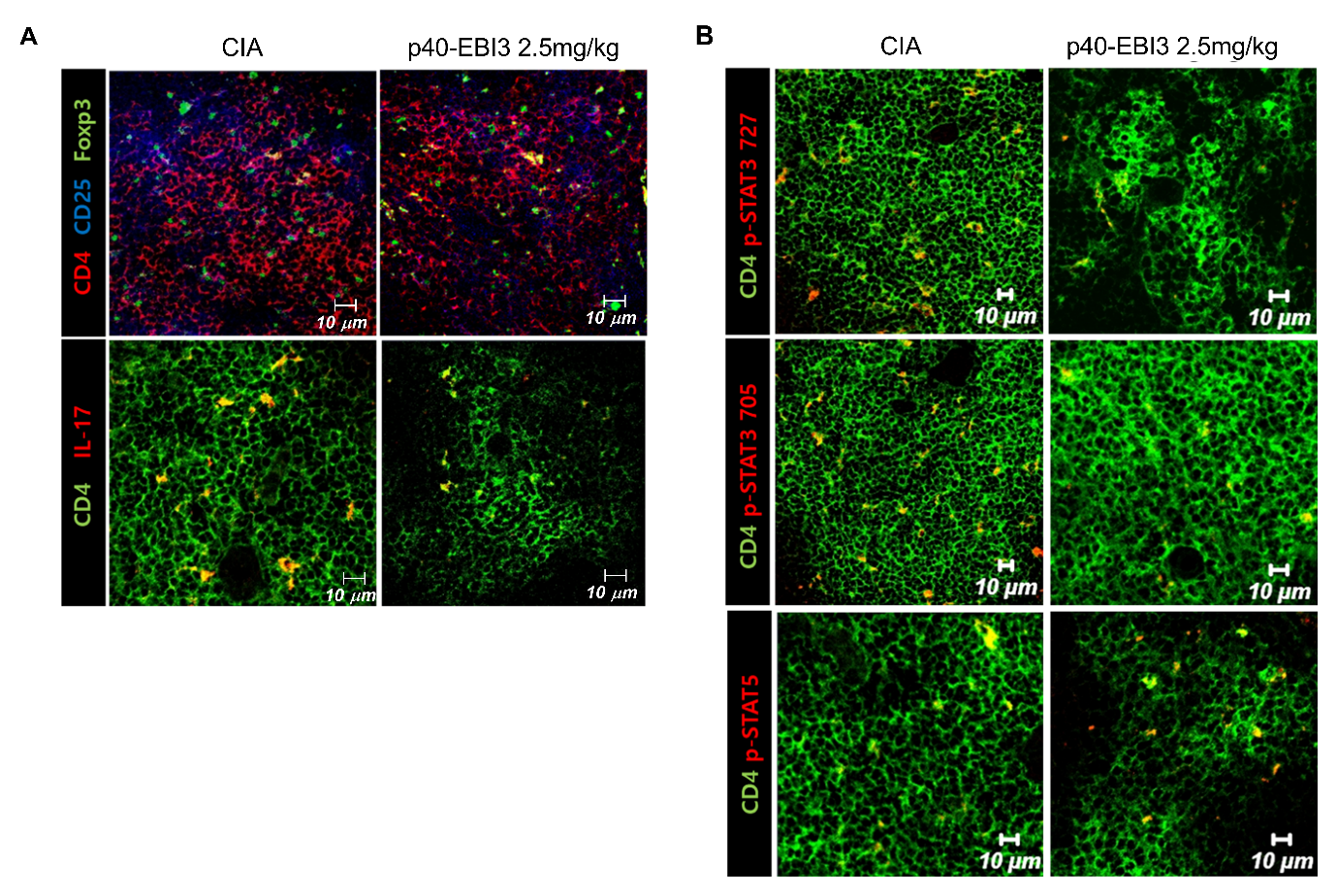
**

**Supplementary Figure 4.** Inhibitory effect of p40-EBI3 on Th17 cell population in a collagen-induced arthritis mouse model. (A) Splenic tissues from each group of mice were stained for CD4^+^CD25^+^Foxp3^+^ Treg cells and CD4^+^IL-17^+^ Th17 cells using monoclonal antibodies against CD4 (red), CD25 (blue), and Foxp3 (green) (*upper image*) or CD4 (green) and IL-17 (red) (*lower image*) (original magnification, ×400). Each confocal image is representative of five fields of view and three separate experiments. (B) Spleens from mice in each group were examined by immunofluorescence staining with monoclonal antibodies against CD4 (green) and pSTAT3 Ser727 (red) (*upper image*), CD4 (green) and pSTAT3 Tyr705 (red) (*middle image*), or CD4 (green) and pSTAT5 (red) (*lower image*). Cell populations were analyzed by laser scanning confocal microscopy (scale bars, 10 μm).


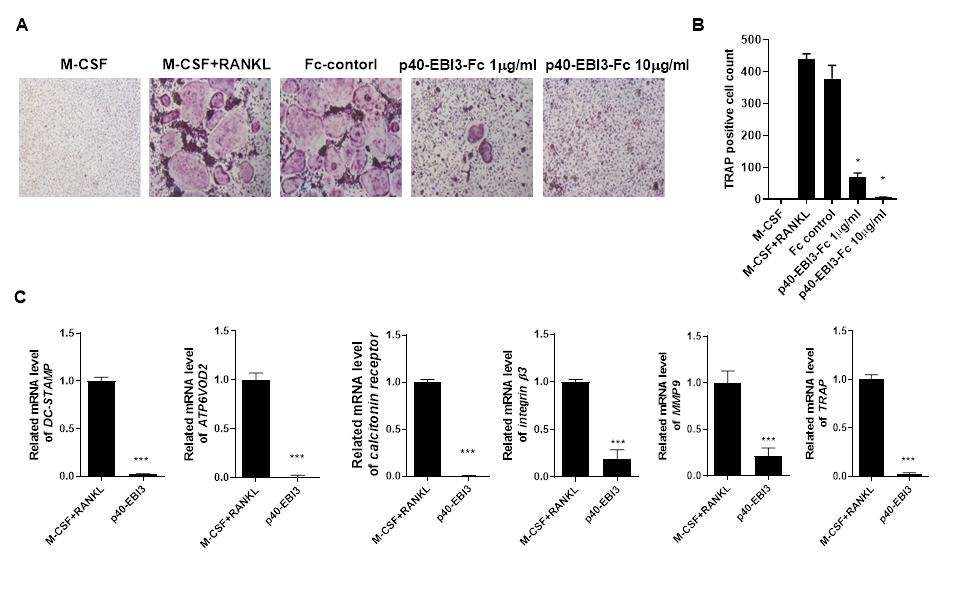


**Supplementary Figure 5.** p40-EBI3-Fc inhibits osteoclast differentiation. (A) Inhibitory effects of p40-EBI3-Fc on *in vitro* osteoclast formation. Naïve murine BMMs were subjected to osteoclast differentiation by combined stimulation with p40-EBI3-Fc, Fc-control, M-CSF, and RANKL as indicated. Photographs (×100) are representative of TRAP staining obtained from three independent experiments. (B) TRAP^+^ osteoclasts (≥ 3 nuclei/TRAP^+^ cell) were counted; values obtained from whole wells of a 48-well plate are presented as the mean ± SD (***p* < 0.01, *** *p* < 0.001 vs. M-CSF and RANKL stimulation condition without p40/EBI3-Fc treatment). (C) Inhibitory effects of p40-EBI3-Fc (10 μg/mL) on osteoclast-specific genes. mRNA levels of *DC-STAMP*, *Atp6v0d2*, *calcitonin receptor*, *integrin β3*, *Mmp9*, and *Trap* were reduced by p40-EBI3-Fc (**p* < 0.05, ****p* < 0.001).


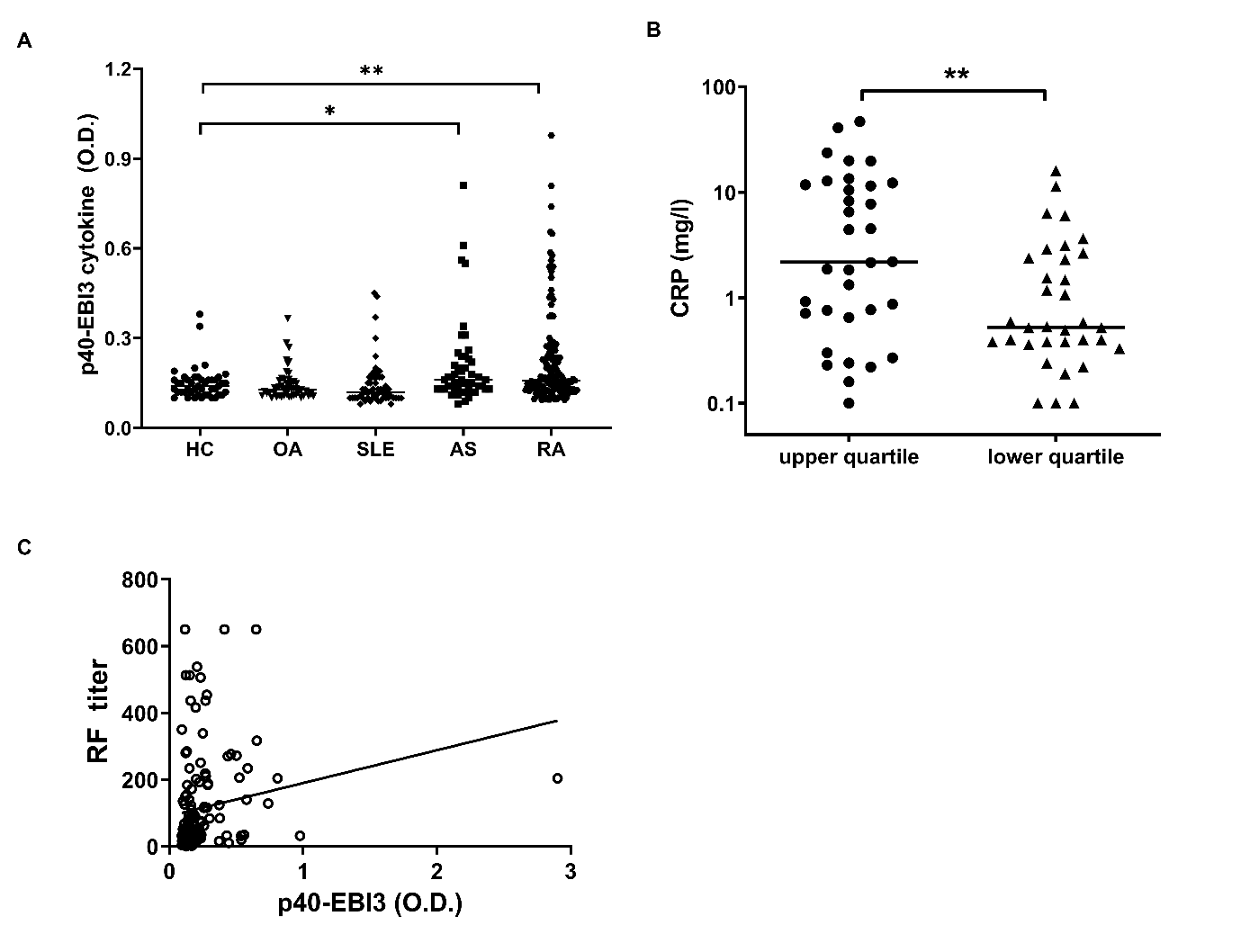


**Supplementary Figure 6.** Serum p40-EBI3 level in patients with RA. A. Serum concentrations of p40-EBI3 in healthy controls (*n* = 47), patients with OA (*n* = 50), patients with SLE (*n* = 60), patients with AS (*n* = 49) and patients with RA (*n* = 136) were measured by ELISA. B. Dot plot showing median CRP levels in two subgroups. Patients with RA in the upper serum p40/EBI3 quartile exhibited significantly higher CRP levels compared to those in the lower quartile. C. Serum p40-EBI3 levels in patients with RA were significantly correlated with RF titer (**p* < 0.05, ***p* < 0.01).


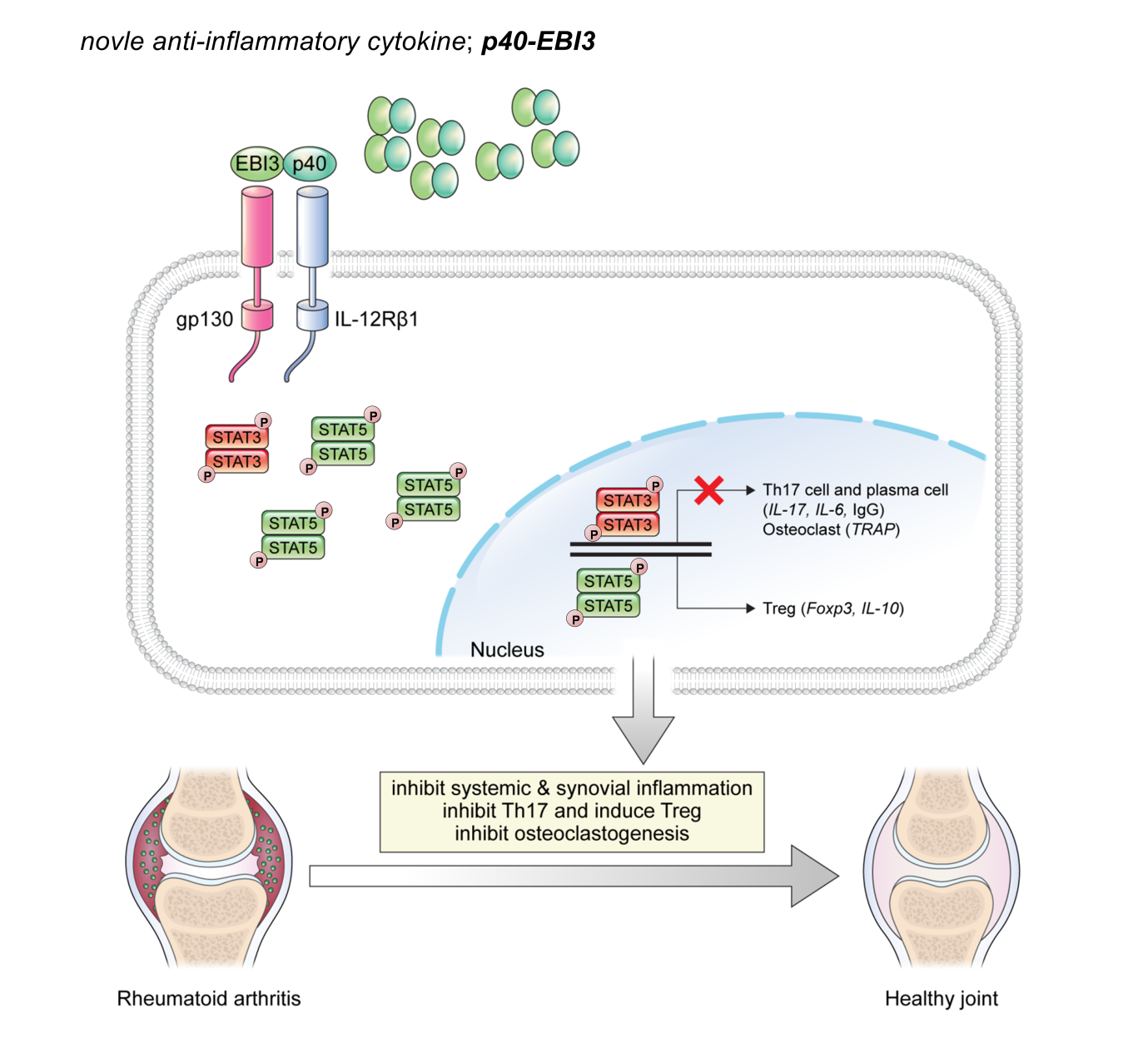


**Supplementary Figure 7.** A novel cytokine consisting of p40 and EBI3 subunits suppresses rheumatoid arthritis.

**References**

1. Arnett FC, Edworthy SM, Bloch DA, McShane DJ, Fries JF, Cooper NS, et al. The American Rheumatism Association 1987 revised criteria for the classification of rheumatoid arthritis. Arthritis Rheum. 1988;31(3):315-24.

2. Altman R, Asch E, Bloch D, Bole G, Borenstein D, Brandt K, et al. Development of criteria for the classification and reporting of osteoarthritis. Classification of osteoarthritis of the knee. Diagnostic and Therapeutic Criteria Committee of the American Rheumatism Association. Arthritis Rheum. 1986;29(8):1039-49.

The English in this document has been checked by at least two professional editors, both native speakers of English. For a certificate, please see:

<http://www.textcheck.com/certificate/rcTUOG>
